# Supplementary material for: Multiomic analyses direct hypotheses for Creutzfeldt-Jakob disease risk genes
Source: Brain. 2025 Jan 27;148(9):3350–63. doi: 10.1093/brain/awaf032 (PMC12404779; doi:10.1093/brain/awaf032)
Supplement: awaf032_Supplementary_Data [file awaf032_supplementary_data.zip › brain-2024-01740-File006.pdf]

Supplementary Material - Multiomic Analyses Direct Hypotheses for Creutzfeldt-Jakob Disease Risk Genes

Fahri Küçükali[1,2,†], Elizabeth Hill[3,†], Tijs Watzeels[1,2], Holger Hummerich[3], Tracy Campbell[3], Lee Darwent[3], Steven Collins[4], Christiane Stehmann[4], Gabor G Kovacs[5], Michael D Geschwind[6], Karl Frontzek[7], Herbert Budka[8], Ellen Gelpi[8], Adriano Aguzzi[7], Sven J van der Lee[9,10,11], Cornelia M van Duijn[12,13], Pawel P Liberski[14], Miguel Calero[15], Pascual Sanchez-Juan[16], Elodie Bouaziz-Amar[17], Jean-Louis Laplanche[17], Stéphane Haïk[18,19], Jean-Phillipe Brandel[18,19], Angela Mammana[20], Sabina Capellari[21], Anna Poleggi[22], Anna Ladogana[22], Dorina Tiple[22], Saima Zafar[23,24], Stephanie Booth[25], Gerard H Jansen[26], Aušrinė Areškevičiūtė[27], Eva Løbner Lund[27,28], Katie Glisic[29], Piero Parchi[20,21], Peter Hermann[23,30], Inga Zerr[23,30], Jiri Safar[29], Pierluigi Gambetti[29], Brian S Appleby[29], John Collinge[3], Kristel Sleegers[1,2], Simon Mead[3*]

[†]These authors contributed equally to this work.

Author affiliations:

[1]Complex Genetics of Alzheimer's Disease group, VIB Center for Molecular Neurology, VIB, Antwerp, Belgium
[2]Department of Biomedical Sciences, University of Antwerp, Antwerp, Belgium
[3]Medical Research Council Prion Unit, University College London Institute of Prion Diseases, London, UK
[4]Australian National Creutzfeldt-Jakob Disease Registry, The Florey and Department of Medicine (RMH), The University of Melbourne, Victoria, 3010, Australia.
[5]Department of Laboratory Medicine and Pathobiology and Tanz Centre for Research in Neurodegenerative Disease, University of Toronto, and Laboratory Medicine Program & Krembil Brain Institute, University Health Network, Toronto, Ontario, Canada
[6]UCSF Memory and Aging Center, Department of Neurology, University of California, San Francisco, USA.
[7]Institute of Neuropathology, University of Zürich, Zürich, Switzerland.
[8] Austrian Reference Centre for Human Prion Diseases, Division of Neuropathology and Neurochemistry, Department of Neurology, Medical University Vienna, Austria.
[9]Genomics of Neurodegenerative Diseases and Aging, Human Genetics, Vrije Universiteit Amsterdam, Amsterdam UMC, location VUmc, Amsterdam, The Netherlands
[10]Alzheimer Center Amsterdam, Neurology, Vrije Universiteit Amsterdam, Amsterdam UMC location VUmc, Amsterdam, The Netherlands
[11]Amsterdam Neuroscience, Neurodegeneration, Amsterdam, The Netherlands
[12]Department of Epidemiology, Erasmus Medical Centre, Rotterdam, The Netherlands
[13]Nuffield Department of Population Health, University of Oxford, UK.
[14]Department of Molecular Pathology and Neuropathology, Medical University of Lodz, Lodz, Poland

[15]Chronic Disease Programme (UFIEC-CROSADIS) and Network Center for Biomedical Research in Neurodegenerative Diseases (CIBERNED), Instituto de Salud Carlos III, Madrid, Spain
[16]Neurology Service, University Hospital Marqués de Valdecilla (University of Cantabria, CIBERNED and IDIVAL), Santander, Spain.
[17]Department of Biochemistry and Molecular Biology, Lariboisière Hospital, GHU AP-HP .Nord, University of Paris Cité, France
[18]Sorbonne Université, INSERM, CNRS UMR 7225, Institut du Cerveau et de la Moelle épinière, ICM, Paris, France
[19]Cellule nationale de référence des maladies de Creutzfeldt-Jakob, AP-HP, University Hospital Pitié-Salpêtrière, Paris, France
[20]IRCCS, Istituto delle Scienze Neurologiche di Bologna, Bologna, Italy.
[21]Department of Biomedical and Neuromotor Sciences, University of Bologna, Bologna, Italy
[22] Department of Neuroscience, Istituto Superiore di Sanità, Roma, Italy.
[23]Department of Neurology, Clinical Dementia Center and National Reference Center for CJD Surveillance, University Medical School, Göttingen, Germany
[24]Biomedical Engineering and Sciences Department, School of Mechanical and Manufacturing Engineering, National University of Sciences and Technology, Islamabad, Pakistan
[25]Prion Disease Program, National Microbiology Laboratory, Public Health Agency of Canada, Winnipeg, Canada
[26]Department of Pathology and Laboratory Medicine, University of Ottawa, Ottawa, Canada.
[27]Danish Reference Center for Prion Diseases, Department of Pathology, Copenhagen University Hospital, Rigshospitalet, Copenhagen 2100, Denmark
[28]Department of Clinical Medicine, University of Copenhagen, Copenhagen, Denmark
[29]National Prion Disease Pathology Surveillance Center, Case Western Reserve University, Cleveland, OH, USA
[30]German Center for Neurodegenerative Diseases (DZNE), Göttingen, Germany

*Correspondence to: Simon Mead

Medical Research Council Prion Unit, University College London Institute of Prion Diseases, London, UK

s.mead@prion.ucl.ac.uk

Supplementary Materials and Methods

Selection of index variants

We define the index variants in this study as the representative variants in each locus reported in the sCJD GWAS publication[1], and the selection of these index variants was carried out based on various criteria, including having the strongest statistical evidence in the discovery phase (the "lead variants", for *PDIA4* and *BMERB1* loci, where *PDIA4* lead variant is also a 3'UTR variant), being the genome-wide significant variants within the coding exons (the missense variants in *PRNP* and *GAL3ST1* loci, and the synonymous variant in STX6 locus) chosen for the targeted replication analyses in the replication stage, and the well-established functional evidence for causality of two of the index variants (rs1799990 for *PRNP* and rs2267161 for GAL3ST1). Importantly, statistical fine-mapping with FINEMAP also listed these 5 index variants in the credible sets for each respective loci (**Supplementary Table 10**). We summarize and discuss functional evidence available for *PRNP* and *GAL3ST1* index variants below.

We prioritised the extensive functional genetics and the biological knowledge base to inform on our choice of the index SNPs included. Compelling evidence over decades has concluded that *PRNP* codon 129 is the causal variant on chromosome 20p13 modulating disease susceptibility and clinical phenotypes[2-9]. Indeed, in the 1990s, it was demonstrated that knockout of *Prnp* in mice conferred complete protection to prion disease[10,11]. Indeed, this established a therapeutic hypothesis which is being pursued by academic and pharmacological entities today by different modalities including *PRNP*-targeting ASOs (Phase 1/2a trial employing *ION717, NCT06153966)* or by employing PrP-targeting antibodies[12]. There are numerous *in vivo*[13-15], cellular[16] and *in vitro* models[17] that demonstrate the profound effects of rs1799990 on the disease biology and strain propagation. Therefore, the clear evidence of a mechanism prioritised rs1799990 as the causal variant.

Similarly, there was clear evidence for a genetic mechanism at the *GAL3ST1* locus with the V29M variant having extensive biological associations in blood lipid metabolites[18,19] and brain structure[20] in human populations providing biological plausibility for this missense variant being causal at the *GAL3ST1* locus.

Statistical fine-mapping with FINEMAP

FINEMAP (v1.4.1), a tool for single locus fine-mapping without using functional annotations, was applied to a +/- 500 kb extended genetic region around the index SNPs (**Supplementary Table 1**) of five loci (*PRNP, STX6, GAL3ST1*, *BMERB1* and *PDIA4*) in the summary statistics of the discovery stage of the sCJD GWAS. We used the shotgun stochastic search algorithm (SSS) with the maximum number of allowed causal SNPs ("--n-causal-snps") option set as 1 in each locus. The LD correlation matrix was generated as an "in-sample LD" matrix using the same samples used to compute the single-SNP association statistics.

Ancillary TWAS analyses

We ran ancillary TWAS analyses with additional expression reference panels (both individual-level and summary-level panels), namely, the large-scale MetaBrain cortex[21], PsychENCODE DLPFC[22], and eQTLGen[23] blood datasets. These results were not included in the main gene prioritization pipeline for various reasons. First, eQTLs in blood are likely to have less direct relevance for CJD than in brain tissue. Second, the TWAS predictors from Pain *et al.* (MetaBrain and eQTLGen reference panels) are based on summary-level eQTL information rather than using the individual-level data typically used in TWAS analyses, which comes with various limitations as the authors discussed[24]. Particularly, eQTLGen blood eQTL summary statistics information originates from 37 cohorts[23] that results in an unstable sample size. Third, the MetaBrain study[21] predominantly overlaps with the TWAS reference panels we used mainly, originating from AMP-AD and GTEx studies, as it combines and meta-analyses the majority of these. Furthermore, a large overlap exists between MetaBrain Cortex and PsychENCODE[22] DLPFC datasets as both include the CommonMind Consortium (CMC), BipSeq, LIBD, UCLA_ASD, CMC_HBCC, and BrainGVEx cohorts (6 out of 8 cohorts included in PsychENCODE), while the CMC cohort includes samples from Mount Sinai brain bank (MSBB), in which we perform TWAS as well. These overlaps could inflate TWAS-derived findings as a false replication, and consequently impact our gene prioritization pipeline as the genes with replicated TWAS hits obtain higher weighted scores (**Supplementary Table 3**). Therefore, these results were not considered towards gene prioritization, however provided for additional potential insights.

Methodologically, we ran individual-level (classical) TWAS using publicly available (see Data Availability in the main manuscript) PsychENCODE DLPFC expression reference panel

(n=1321 samples) provided in GRCh37 human reference genome assembly (in combination with sCJD GWAS summary statistics originally available in GRCh37 as well), and we considered TWAS significance based on number of genes tested (n=7564, corresponding to a Bonferroni-corrected significance threshold of 6.6×10^{-6}). Next, we ran summary-level TWAS in MetaBrain and eQTLGen using the reference panels provided in Pain *et al.*[24], which used summary-level eQTL information to construct summary-level TWAS reference panels with seven distinct prediction models ("top1", "susie", "sbayesr", "sbayesr_robust", "prscs", "ldpred2", "lassosum", and "dbslmm"; see Pain *et al.*[24]). We ran MetaBrain cortex (n=2743 samples) and eQTLGen blood (n_{max}=31684 samples) summary-level TWAS using the heritable (heritability P, "hsq_p", < 0.05) features in these prediction models, and we defined the Bonferroni-corrected significance thresholds by the number of tests performed in each summary-level prediction models (significance threshold ranging between 4.25×10^{-6} and 3.1×10^{-6}).

Tier classification in the gene prioritization pipeline

The gene prioritization tier classification methodology used in this study is adapted from our previous study[25], in which the procedure is explained in detail. Briefly, after obtaining a total weighted score per gene, we ranked genes per locus according to their gene prioritization scores and compared the relative score differences between the highest ranked gene and other genes in each investigated locus. If this relative difference was at least 20% and the gene prioritization score for the highest ranked gene was ≥4, then we classified this gene as a tier 1 prioritized gene in the investigated locus. If this absolute threshold was not met, then the highest ranked gene was classified as a tier 2 prioritized gene (i.e., a lower level of confidence due to absence of the minimum level of evidence for a true risk gene we consider). Moreover, other genes in a locus harboring a tier 1 gene were classified as tier 2 prioritized genes if the relative score difference versus the highest ranked (tier 1) gene was between 20% and 50%. Finally, when the relative score difference between the highest ranked gene and other genes in the same locus was <20%, then both the highest ranked gene and all genes with a score difference <20% were classified as tier 2 prioritized genes in the investigated locus; meaning that it is difficult to differentiate and prioritize two or more similarly scored genes based on the current evidence[25].

Supplementary Results

Ancillary TWAS results

While not used in main gene prioritization pipeline, we identified several potentially interesting significant results (**Supplementary Table 11**) in ancillary TWAS analyses we ran in PsychENCODE DLPFC, MetaBrain cortex, and eQTLGen blood. First, our *STX6* TWAS finding was replicated significantly and in the same directionality in both PsychENCODE DLPFC and in MetaBrain cortex panels; in addition to the replication of *PDIA4* in MetaBrain cortex, further supporting their prioritization. Second, MetaBrain cortex listed two other significant new genes as TWAS-significant genes: *DRG1* and *SLC9A5*. Third, eQTLGen blood TWAS identified 29 genes, among which we identified the first TWAS evidence for *PRNP* in eQTLGen blood, with negative Z-score, meaning that the sCJD genetic risk was negatively correlated with predicted expression of *PRNP* in blood. (**Supplementary Table 11**).

References

1. Jones E, Hummerich H, Vire E, *et al*. Identification of novel risk loci and causal insights for sporadic Creutzfeldt-Jakob disease: a genome-wide association study. *Lancet Neurol*. Oct 2020;19(10):840-848. doi:10.1016/S1474-4422(20)30273-8
2. Collinge J, Palmer MS, Dryden AJ. Genetic predisposition to iatrogenic Creutzfeldt-Jakob disease. *The Lancet*. 1991;337(8755):1441-1442. doi:10.1016/0140-6736(91)93128-V
3. Collinge J, Whitfield J, McKintosh E, *et al*. Kuru in the 21st century--an acquired human prion disease with very long incubation periods. *Lancet*. Jun 24 2006;367(9528):2068-74. doi:10.1016/S0140-6736(06)68930-7
4. Minikel EV, Vallabh SM, Orseth MC, *et al*. Age at onset in genetic prion disease and the design of preventive clinical trials. *Neurology*. Jul 9 2019;93(2):e125-e134. doi:10.1212/wnl.0000000000007745
5. Mead S, Poulter M, Beck J, *et al*. Inherited prion disease with six octapeptide repeat insertional mutation--molecular analysis of phenotypic heterogeneity. *Brain*. Sep 2006;129(Pt 9):2297-317. doi:10.1093/brain/awl226
6. Palmer MS, Dryden AJ, Hughes JT, Collinge J. Homozygous prion protein genotype predisposes to sporadic Creutzfeldt–Jakob disease. *Nature*. 1991/07/01 1991;352(6333):340-342. doi:10.1038/352340a0
7. Webb TE, Whittaker J, Collinge J, Mead S. Age of onset and death in inherited prion disease are heritable. *Am J Med Genet B Neuropsychiatr Genet*. Jun 5 2009;150B(4):496-501. doi:10.1002/ajmg.b.30844
8. Webb TE, Poulter M, Beck J, *et al*. Phenotypic heterogeneity and genetic modification of P102L inherited prion disease in an international series. *Brain*. Oct 2008;131(Pt 10):2632-46. doi:10.1093/brain/awn202

9. Mok T, Jaunmuktane Z, Joiner S, *et al*. Variant Creutzfeldt–Jakob Disease in a Patient with Heterozygosity at PRNP Codon 129. *New England Journal of Medicine*. 2017/01/19 2017;376(3):292-294. doi:10.1056/NEJMc1610003
10. Bueler H, Aguzzi A, Sailer A, *et al*. Mice devoid of PrP are resistant to scrapie. *Cell*. Jul 2 1993;73(7):1339-47. doi:10.1016/0092-8674(93)90360-3
11. Sailer A, Bueler H, Fischer M, Aguzzi A, Weissmann C. No propagation of prions in mice devoid of PrP. *Cell*. Jul 1 1994;77(7):967-8. doi:10.1016/0092-8674(94)90436-7
12. Mead S, Khalili-Shirazi A, Potter C, *et al*. Prion protein monoclonal antibody (PRN100) therapy for Creutzfeldt-Jakob disease: evaluation of a first-in-human treatment programme. *Lancet Neurol*. Apr 2022;21(4):342-354. doi:10.1016/S1474-4422(22)00082-5
13. Wadsworth JD, Asante EA, Desbruslais M, *et al*. Human prion protein with valine 129 prevents expression of variant CJD phenotype. *Science*. Dec 3 2004;306(5702):1793-6. doi:10.1126/science.1103932
14. Takeuchi A, Kobayashi A, Ironside JW, Mohri S, Kitamoto T. Characterization of variant Creutzfeldt-Jakob disease prions in prion protein-humanized mice carrying distinct codon 129 genotypes. *The Journal of biological chemistry*. Jul 26 2013;288(30):21659-66. doi:10.1074/jbc.M113.470328
15. Asante EA, Linehan JM, Gowland I, *et al*. Dissociation of pathological and molecular phenotype of variant Creutzfeldt-Jakob disease in transgenic human prion protein 129 heterozygous mice. *Proc Natl Acad Sci U S A*. Jul 11 2006;103(28):10759-64. doi:10.1073/pnas.0604292103
16. Krejciova Z, Alibhai J, Zhao C, *et al*. Human stem cell–derived astrocytes replicate human prions in a PRNP genotype–dependent manner. *Journal of Experimental Medicine*. 2017;214(12):3481-3495. doi:10.1084/jem.20161547
17. Pham N, Yin S, Yu S, *et al*. Normal cellular prion protein with a methionine at position 129 has a more exposed helix 1 and is more prone to aggregate. *Biochemical and biophysical research communications*. 2008/04/18/ 2008;368(4):875-881. doi:https://doi.org/10.1016/j.bbrc.2008.01.172
18. Cadby G, Giles C, Melton PE, *et al*. Comprehensive genetic analysis of the human lipidome identifies loci associated with lipid homeostasis with links to coronary artery disease. *Nature Communications*. 2022/06/06 2022;13(1):3124. doi:10.1038/s41467-022-30875-7
19. Harshfield EL, Fauman EB, Stacey D, *et al*. Genome-wide analysis of blood lipid metabolites in over 5,000 South Asians reveals biological insights at cardiometabolic disease loci. *medRxiv*. 2020:2020.10.16.20213520. doi:10.1101/2020.10.16.20213520
20. Zhao B, Zhang J, Ibrahim JG, *et al*. Large-scale GWAS reveals genetic architecture of brain white matter microstructure and genetic overlap with cognitive and mental health traits (n = 17,706). *Molecular Psychiatry*. 2019/10/30 2019;doi:10.1038/s41380-019-0569-z
21. de Klein N, Tsai EA, Vochteloo M, *et al*. Brain expression quantitative trait locus and network analyses reveal downstream effects and putative drivers for brain-related diseases. *Nat Genet*. Mar 2023;55(3):377-388. doi:10.1038/s41588-023-01300-6
22. Gandal MJ, Zhang P, Hadjimichael E, *et al*. Transcriptome-wide isoform-level dysregulation in ASD, schizophrenia, and bipolar disorder. *Science*. Dec 14 2018;362(6420)doi:10.1126/science.aat8127
23. Vosa U, Claringbould A, Westra HJ, *et al*. Large-scale cis- and trans-eQTL analyses identify thousands of genetic loci and polygenic scores that regulate blood gene expression. *Nat Genet*. Sep 2021;53(9):1300-1310. doi:10.1038/s41588-021-00913-z
24. Pain O, Gerring Z, Derks E, Wray N, Gusev A, Al-Chalabi A. Polygenic Prediction of Molecular Traits using Large-Scale Meta-analysis Summary Statistics. *bioRxiv*. 2022 2022;doi:10.1101/2022.11.23.517213

25. Bellenguez C, Küçükali F, Jansen IE, *et al*. New insights into the genetic etiology of Alzheimer's disease and related dementias. *Nature Genetics*. 2022/04/01 2022;54(4):412-436. doi:10.1038/s41588-022-01024-z
